# A new genus of Asiatic short-tailed shrew (Soricidae, Eulipotyphla) based on molecular and morphological comparisons

**DOI:** 10.24272/j.issn.2095-8137.2018.058

**Published:** 2018-06-28

**Authors:** Kai He, Xing Chen, Peng Chen, Shui-Wang He, Feng Cheng, Xue-Long Jiang, Kevin L. Campbell

**Affiliations:** 1 Kunming Institute of Zoology, Chinese Academy of Sciences, Kunming Yunnan 650223, China; 2 Department of Biological Sciences, University of Manitoba, Winnipeg, Manitoba R3T 2N2, Canada

**Keywords:** Blarinellini, Capture-hybridization, Mitogenome, Molecular phylogeny, Next-generation sequencing, *Pantherina*

## Abstract

Blarinellini is a tribe of soricine shrews comprised of nine fossil genera and one extant genus. Blarinelline shrews were once widely distributed throughout Eurasia and North America, though only members of the Asiatic short-tailed shrew genus *Blarinella* currently persist (mostly in southwestern China and adjacent areas). Only three forms of *Blarinella* have been recognized as either species or subspecies. However, recent molecular studies indicated a strikingly deep divergence within the genus, implying the existence of a distinct genus-level lineage. We sequenced the complete mitochondrial genomes and one nuclear gene of three Asiatic short-tailed and two North American shrews and analyzed them morphometrically and morphologically. Our molecular analyses revealed that specimens ascribed to *B. griselda* formed two deeply diverged lineages, one a close relative to *B. quadraticauda*, whereas the other—comprised of topotype specimens from southern Gansu—diverged from other *Blarinella* in the middle Miocene (ca. 18.2 million years ago (Ma), 95% confidence interval=13.4–23.6 Ma). Although the skulls were similarly shaped in both lineages, we observed several diagnostic characteristics, including the shape of the upper P^4^. In consideration of the molecular and morphological evidence, we recognize *B. griselda* as the sole species of a new genus, namely, *Pantherina* **gen. nov.** Interestingly, some characteristics of *Pantherina griselda* are more similar to fossil genera, suggesting it represents an evolutionarily more primitive form than *Blarinella*. Recognition of this new genus sheds light on the systematics and evolutionary history of the tribe Blarinellini throughout Eurasia and North America.

## INTRODUCTION

Asiatic short-tailed shrews, currently classified as species in the genus *Blarinella*, are small insectivorous mammals distributed mainly in central and southwestern China, adjacent Myanmar, and northern-most Vietnam. These small- to middle-sized shrews are uniformly black or dark brown and have large incisors, heavy tooth pigmentation, and a short tail that is typically 40%–60% of the head-body length. The fore claws are enlarged, suggesting adaptation for a semi-fossorial lifestyle (Wilson & Mittermeier, 2018).

The taxonomy of *Blarinella* has been studied since the late 19^th^ century. The first recognized species was *Sorex quadraticauda*, described by Milne-Edwards & Milne-Edwards (1872) based on a specimen from Baoxing (=Mouping), northwestern Sichuan, China (the same type locality as the giant panda, *Ailuropoda melanoleuca* (David, 1869)). Milne-Edwards & Milne-Edwards (1872) documented the shrew’s relatively short and somewhat square-shaped tail, well-developed incisors, and intensively dark pigmentation on the teeth. This species typically has five upper unicuspids, although the holotype specimen has only four (with the fifth one missing) on one side of its skull and five on the other, as discussed by Thomas (1911). When Thomas (1911) examined new specimens from Mt. Emei (Omi-San, 100 km south of Baoxing) in western Sichuan, *S*. *quadraticauda* was determined to be more closely related to the North American short-tailed shrews *Blarina* and *Cryptotis* (tribe Blarinini), than to the Old World *Sorex*, and thus assigned to its own genus *Blarinella* (literally “small *Blarina*”).

Thomas himself recognized additional two *Blarinella* species. One (*Blarinella griselda*) was based on specimens from Lintan (=Taochou), Gansu, which were differentiated by their smaller size, grayer color, and shorter tails (Thomas, 1912). Thomas (1915) later recognized specimens from Pianma (=Hpimaw), Yunnan, as a third species, *Blarinella wardi*, based on their small size, dark color, and narrow skull. Since then, specimens collected across southern China and northern-most Vietnam have been assigned to one of these three groupings, though their taxonomic status has varied. For example, Allen (1938) and Hutterer (1993) placed *B. griselda* and *B. wardi* as subspecies in *B. quadraticauda*, whereas Corbet (1978) and Hutterer (2005) recognized all three as distinct species.

Jiang et al. (2003) reviewed the taxonomy and distribution of the group and, based on multivariate and univariate morphometric analyses of skulls, recognized the three as distinct species but assigned only a few populations from northwestern Sichuan to *B. quadraticauda*. This three-species division has been widely accepted (Hutterer, 2005; Smith & Xie, 2008). Chen et al. (2012) was the first to apply molecular phylogenetic approaches to samples collected mostly from western Sichuan and Shaanxi. Their study revealed *B. wardi* as a distinct lineage at a basal position of the genus, confirming its species status, and found *B. quadraticauda* was a well-supported clade embedded within *B. griselda*, making the latter a paraphyletic group. The authors suggested that either this represented an incipient speciation event for *B. quadraticauda,* or the taxonomy of the genus was incorrect. It is worth noting that no holotype or topotype specimens of *B. griselda* from southern Gansu were included in either study, so the populations from Chongqing, Hubei, and eastern Yunnan were tentatively assigned to *B. griselda* based on their intermediate size between *B. quadraticauda* and *B. wardi*.

All the studies mentioned above examined *Blarinella* taxonomy at the species/subspecies levels, implicitly accepting the monophyly of the genus. More recently, however, He (2011) discovered two genetically distinct, but sympatrically distributed, lineages from Mt. Qinling, Shaanxi, China, that called this into question. One lineage clustered with previously sequenced *Blarinella griselda*, whereas the other formed a cryptic lineage that appears to have diverged from other *Blarinella* more than 10 million years ago (Ma), suggesting that an undescribed genus may exist. However, because the Mt. Qinling and southern Gansu mountains (including Lintan, the type locality of *B. griselda*) are on the same tectonic Qinling belt, and because no specimen from Gansu has ever been included in morphometric or genetic study, it was uncertain whether the cryptic lineage represented *B. griselda* or an unrecognized taxon. Despite efforts to explore the southern Gansu mountains (see He et al., 2013), no *Blarinella* species have been captured, and the question remains unresolved. Based on sequences from a single specimen from Gansu and a handful of specimens from Vietnam, Bannikova et al. (2017) confirmed the existence of two clades in the genus *Blarinella*. Because their sample locality in Gansu was near the type locality of *B. griselda*, their specimen is likely to be the “true” *B. griselda*. Based on the results of these two studies, we hypothesized that *B. griselda* occurs only in Gansu and Shaanxi. We further hypothesized that this distinct evolutionary lineage may represent a separate genus, and that “*B. griselda*” from central and southern China, northern Vietnam, and Mt. Qinling is likely to be more closely related to *B. quadraticauda*. Thus, a revision of the taxonomic status of *B. griselda* is warranted.

The systematic position of *Blarinella* has been revised by paleontologists based on craniodental characteristics and revisited by mammalogists using molecular data. Repenning (1967) firstly included the genus in the tribe Soricini, despite acknowledging the similarity between *Blarinella* and Blarinini (North American *Blarina* and *Cryptotis*). Reumer (1998) established the new tribe Blarinellini to include the extant *Blarinella* plus eight fossil genera. A close relationship between Blarinini and Blarinellini is supported by molecular phylogenetic analyses (Dubey et al., 2007; He et al., 2010). These lines of evidence together point to *Blarinella* as a relict genus of Blarinellini.

The craniodental characteristics of *Blarinella* were first described by Repenning (1967) based on *B. quadraticauda* and fossil species *B. kormosi* (=*Zelceina kormosi*; Storch, 1995). Several characters recognized by Repenning (1967) were adopted by Reumer (1998) for defining the tribe Blarinellini, such as breadth of the interarticular area on the mandibular condyle, reduced posterior emargination on the upper molariform teeth, presence of entoconid crests on M_1_ and M_2_, and reduction of the talonid of M_3_.

In the current study, we sequenced five complete mitochondrial genomes and seven partial *ApoB* genes of two *Blarinella* and five *Blarina* specimens and also compared their craniodental morphology. We used these data to update the taxonomy of *Blarinella*, focusing on the distinctiveness of *B. griselda* from Gansu and Shaanxi.

## MATERIALS AND METHODS

### Sampling and experiments

**Figure 1:**
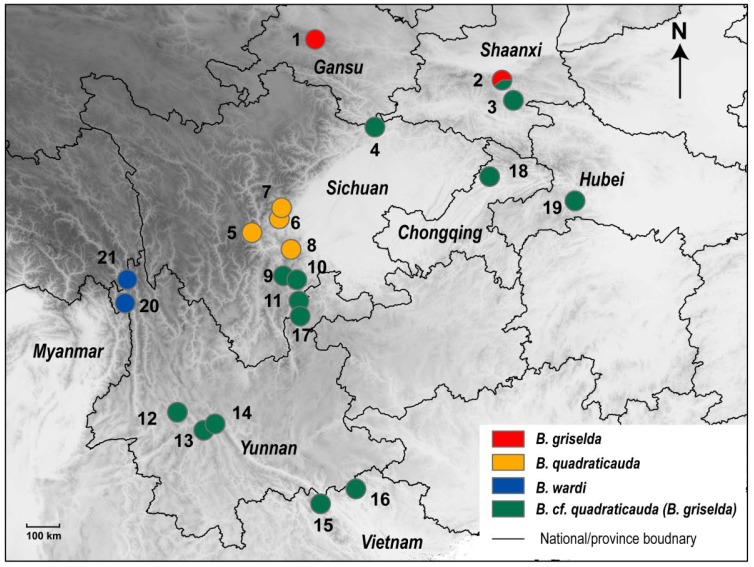
Sample localities of specimens used for molecular analyses

All specimens and tissue samples were obtained following locally approved animal care procedures under the auspices of authorized collection permits. *Blarinella* specimens from China were collected by the Mammal Ecology and Evolution Group of the Kunming Institute of Zoology, Chinese Academy of Sciences, from Mts. Ailao, Gongshan, and Qinling (Figure 1). *Blarina brevicauda* was collected near Bird Lake in Nopiming Provincial Park, Manitoba, Canada, while conducting research under Manitoba Conservation Permit WB12563. A tissue sample of *Blarina hylophaga* was obtained from the National Museum of Natural History, Smithsonian Institution, Washington DC, USA (USNM 568994) under transaction No. 2073785 (Supplementary Table S1). DNA was extracted using a DNA extraction kit (Qiagen DNeasy Blood & Tissue Kits, China) or the phenol/proteinase K/sodium dodecyl sulphate method (Sambrook et al., 2001). For each specimen, we sequenced the complete mitochondrial cyt *b* gene and a nuclear *ApoB* gene segment using Sanger sequencing. The primers were developed in previous studies (Dubey et al., 2007; He et al., 2010) and are given in Supplementary Table S2. Each PCR product was sequenced using both the sense and anti-sense primers and were assembled using Lasergene SeqMan v7 (DNASTAR).

*Blarinella* cf. *quadraticauda* (green dots) represents localities for specimens previously identified as *B. griselda*. Numbers refer to sample localities provided in the supplementary file.

We selected one sample per species for *Blarina brevicauda*, *Blarina hylophaga*, and *Blarinella wardi*, and one for each of the two distinct lineages of *B. griselda* for sequencing of the complete mitochondrial genomes (mitogenome(s) hereafter) using next-generation sequencing (NGS). We used two approaches to obtain the mitogenomes, that is, long-range PCR and cross-species hybridization capture. Our full protocol has been described and published separately (Chen et al., 2018). In short, we first amplified the mitogenomes using Phusion High-Fidelity DNA Polymerase (New England Biolabs, Canada) with two pairs of primers (Supplementary Table S2) designed within conserved regions of *12s rRNA*, *16s rRNA*, *COX3*, and *ND1*. The amplicons (~8 000 and 10 000 bp in length) were purified using Serapure magnetic beads (Rohland & Reich, 2012) sheared to small fragments using NEBNext dsDNA Fragmentase (New England Biolabs, Canada) and ligated with barcode adapters (NEXTflex DNA Barcodes for Ion Torrent, BIOO Scientific, USA) using a NEBNext Fast DNA Library Prep Set for Ion Torrent (New England Biolabs, Canada) to construct DNA libraries. After ligation, the libraries were purified using Serapure magnetic beads and size-selected using a 2% E-gel on an E-Gel Electrophoresis System (Invitrogen, Canada). Libraries within the 450–500 bp size range were selected and re-amplified using a NEBNext High-Fidelity 2X PCR Master Mix (New England Biolabs, Canada). We then purified the libraries using Serapure magnetic beads and measured the DNA concentration of the purified libraries using Qubit Fluorometric Quantitation (Thermo Fisher Scientific, Canada).

In cases where the sample could not be amplified successfully using the primers, we used an in-solution capture-hybridization approach to enrich mitochondrial DNA (Horn, 2012; Mason et al., 2011). Briefly, this approach uses mitochondrial probes and DNA libraries constructed using genomic DNA to capture mitochondrial-like sequences. To make the probes, we first amplified and purified two amplicons of the *Blarinella griselda* mitogenome. We then measured the DNA concentration of each amplicon using a Nanodrop 2000 spectrophotometer (Thermo Fisher Scientific, Canada) and mixed the two amplicons to ensure the amount of DNA was in proportion to their relative lengths. The mixed DNA was used to make mitogenome probes (hereafter termed baits) using a Biotin-Nick Translation mix kit (Roche, Germany) according to the manual. The baits were stored at –20 ${}^{\circ }$C before hybridization.

We constructed 450–500 bp-size libraries for each sample from previously extracted genomic DNA (each with a unique barcode as described above) using a NEBNext Fast DNA Library Prep Set for Ion Torrent (New England Biolabs, Canada). The libraries were re-amplified and purified (see above). We mixed the baits and each DNA library at a ratio of approximately 1:10 and then incubated them for 24–48 h at 65 ${}^{\circ }$C (Chen et al., 2018). The enriched libraries were reamplified and quantified using a Qubit as described above. Finally, we pooled the samples to ensure that each had a similar amount of DNA. We sequenced the samples using a v318 chip with the Ion Torrent Personal Genome Machine (PGM).

### Molecular data processes and analyses

After sequencing, we first binned the samples based on their sample-specific barcode adapters and converted the raw reads to FASTQ format using the Torrent Suite v4.0.2 (Thermo Fisher Scientific, Canada). We filtered out reads shorter than 60 bp and trimmed off adapter sequences before assembly. We used MIRA4 for *de novo* assemblies (Chevreux et al., 1999), during which we used a strategy (technology=iontor) specifically suitable for ion torrent data to better resolve the homopolymer insertions-deletions problem (Bragg et al., 2013). We also used the Geneious iterative approach (up to 100 iterations) to map the reads to a reference mitogenome of *Blarinella quadraticauda* from Baoxing, Sichuan, China (GenBank accession No. KJ131179.1). These assemblies were conducted using Geneious v8.1 (Kearse et al., 2012). We repeated both the MIRA4 and Geneious assemblies at least twice for each sample, aligned the assemblies for each sample using MUSCLE (Edgar, 2004), and generated a 50% consensus sequence in Geneious v8.1. We also compared the assembled mitogenome with the cyt *b* sequence of the same sample conducted using Sanger sequencing to ensure the mismatch between the two was ≤1 bp per 1 000 bp for quality control. Finally, we annotated the mitogenomes using the annotation function in Geneious v8.1. We removed the repeat region of the D-loop region assuming the short NGS reads were not long enough to correctly assemble.

For phylogenetic analyses, we downloaded available mitogenomes of 13 soricine and one crocidurine (*Crocidura attenuata*) shrew from GenBank. We also included *16S rRNA* and cyt *b* fragments of *Blarinella*, most of which were collected in one previous study (Chen et al., 2012), for a mitochondrial gene tree estimation. We aligned the mitogenomes with partial *16S rRNA* and cyt *b* using MUSCLE and removed all tRNAs, the *ND6* gene (which is on the light chain), and the D-loop region from the alignment. The remaining 13 375-bp alignment, which included all coding genes on the heavy chain and two rRNA genes, was used for maximum-likelihood (ML) tree estimation using RAxML (Stamatakis, 2014) and implemented in CIPRES (Miller et al., 2010). We subdivided the alignment by genes (15 partitions) and employed a GTR+G model for each gene. We conducted rapid bootstrapping and searched for the best-scoring ML tree, allowing the program to halt bootstrapping automatically under an extended majority rule criterion (autoMRE). We also subdivided the alignment by gene and codon positions (for coding genes) into 38 data blocks and conducted an additional analysis for comparison.

We estimated an *ApoB* gene tree to ensure the mitogenomic tree was not strongly affected by incomplete lineage sorting or mitochondrial introgression. To accompany our newly collected sequences, we obtained sequences from previous studies (Dubey et al., 2007; He, 2011) and downloaded sequences of 10 soricid shrews as outgroups (Supplementary Table S1). We estimated the RAxML *ApoB* gene tree as described above. The *ApoB* gene was considered as a single partition because few mutations were observed in *Blarinella* sequences of the alignment.

We estimated lineage divergence times from the mitogenome data. While we recognize that mitochondrial genes may overestimate true divergence times, this effect can be mitigated using appropriate calibrations. We applied a second calibration following Springer et al. (2018), which focused on the divergence times of eulipotyphlans, and estimated that the divergence between Crocidurinae and Soricinae occurred 36 Ma (95% confidence interval (CI)=28.6–44.0 Ma). This is much older than fossil calibration used in previous studies (Dubey et al., 2007; He et al., 2010), which was based on the assumption that Crocidosoricinae was the ancestor of Crocidurinae, Myosoricinae, and Soricinae. Crocidosoricinae is now recognized as a tribe of Myosoricinae (Crocidosoricini), thereby invalidating that assumption (Furio et al., 2007). Fossils of both crocidurines and soricines are known from the Oligocene (<34 Ma), with the oldest soricid fossil, *Soricolestes soricavus*, found from Middle Eocene strata, Khaychin Formation (Lopatin, 2002). Thus, the estimated divergence time in Springer et al. (2018) is congruent with fossil records. We estimated divergence times using BEAST v2.5 (Bouckaert et al., 2014). We first estimated the best partition scheme and evolutionary model for each partition using PartitionFinder v2.1.1 (Lanfear et al., 2017). A four-partition scheme (Supplementary Table S3) was selected and the GTR+G model was supported as the best-fitting model for each partition. The monophyly of Soricinae was constrained so that *Crocidura* was fixed at the root of the tree. We employed the Birth-Death model as the tree prior and relaxed lognormal as the clock model prior. We used lognormal distribution for the prior of the divergence time of Soricidae. We set the mean to 36 Ma, with a standard deviation of 0.135, so that the median of the prior was 35.7 Ma and the 95% CI was 28.6–44.5 Ma. We set 4×10^8^ generations for each analysis and sampled every 4 000 generations. Convergence was assessed using Tracer v1.7 (Rambaut et al., 2018). We repeated the analyses twice and combined the tree files using LogCombiner v2.5 and estimated the maximum clade credibility tree using Tree Annotator v2.5, both in the BEAST v2.5 package.

### Specimen examination and morphometric analyses

We recorded morphological measurements from five newly collected *Blarinella* specimens from Shaanxi (representing the two distinct lineages of *B. griselda*), and included specimens used in our previous study, which were mainly deposited in the American Museum of Natural History, Smithsonian National Museum of Natural History, and Kunming Institute of Zoology (Appendix I; Jiang et al., 2003). External measurements, including head and body length (HB), tail length (TL), and hind foot length (HF), were recorded from specimen labels or field notes. Nineteen craniomandibular variables were measured using a digital caliper graduated to 0.01 mm: condyloincisive length (CIL), interorbital breadth (IOB), cranial breadth (CB), cranial height (CH), maxillary breadth (MB), rostral length (RL), postrostral length (PRL), palatoincisive length (PIL), postpalatal length (PPL), upper toothrow length (UTR), maximum width across upper second molars (M2-M2), postpalatal depth (PPD), mandibular length (ML), lower toothrow length (LTR), length of lower incisor (LLI), zygomatic plate breadth (ZP), condyle-glenoid length (CGT), and breadth of coronoid process (BCP). We analyzed morphometric variation using craniomandibular variables for 88 intact skulls by principal component analyses (PCA). We log_10_-transformed each variable prior to conducting the PCA in SPSS v17.0 (SPSS Inc, Chicago, Illinois, USA). Based on the results of our molecular analyses (see Results), we assigned the single individual from Gansu and three of the Shaanxi specimens to *B. griselda*; the two other Shaanxi specimens previously identified as *B. griselda* were tentatively identified as *B*. cf. *quadraticauda*.

### Morphological comparison and diagnosis

We examined the morphology of the specimens as per Jiang et al. (2003), Repenning (1967), Reumer (1984), and Storch (1995), and followed their terminology for morphological descriptions. We took photos of skulls and teeth using a digital microscope VHX-2000C (KEYENCE).

## RESULTS

### Phylogenetic relationships

**Figure 2:**
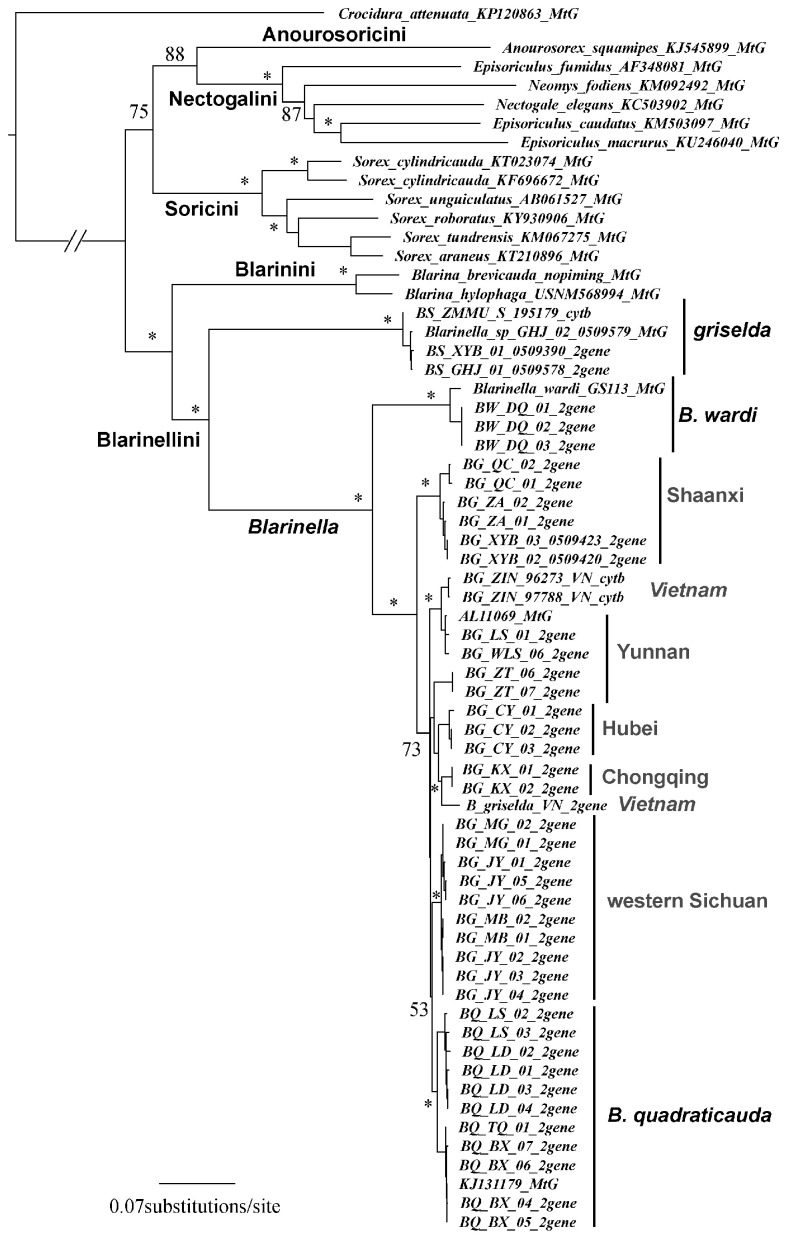
Mitochondrial gene tree

Regardless of the partitioning scheme, each of our mitochondrial datasets revealed the same relationships with similar bootstrap values (BS); thus, only the tree partitioned by genes is presented (Figure 2). Nectogaline (*Episoriculus*, *Neomys*, and *Nectogale*) + anourosoricine (*Anourosorex*) shrews were strongly supported as a clade (BS=88) placed sister to the Soricini with moderate support (BS=75). Species of the genus *Episoriculus* were recovered as paraphyletic (BS=87). The Blarinellini (*Blarinella*) and Blarinini (*Blarina*) were strongly supported as a clade (BS=100), but, within Blarinellini, *Blarinella* formed two distinct sister clades (BS=95), the branch lengths of which were as long as those between Anourosoricini and Nectogalini. One of the two *Blarinella* clades contained the three specimens from Shaanxi and the single specimen from Gansu that we identified as *B. griselda*. Within the second major *Blarinella* clade, four specimens of *B. wardi* from northwestern Yunnan (Gongshan and Zhongdian) formed a distinct branch (BS=100) sister to the subclade consisting of the remaining specimens from Sichuan, Shaanxi, Yunnan, Chongqing, Hubei, and North Vietnam, previously identified as either *B. quadraticauda* or *B. griselda* (BS=100). There were several strongly supported subdivisions in the latter subclade. For example, samples from southern Shaanxi and northeastern-most Sichuan (Qingchuan, adjacent to Shaanxi; Figure 1) formed a basal position, sister to other members of this subclade (BS=73). Samples from Vietnam were placed in two distinct lineages (BS=100).

Branch lengths represent substitutions per site. Numbers above branches are bootstrap values supporting the relationship. Asterisks indicate bootstrap value is higher than 90. Key to type of gene data: 18MtG: mitogenome; 42 2 gene:16S rRNA and cyt *b*; 3 cyt *b*: only cyt *b* gene.

**Figure 3:**
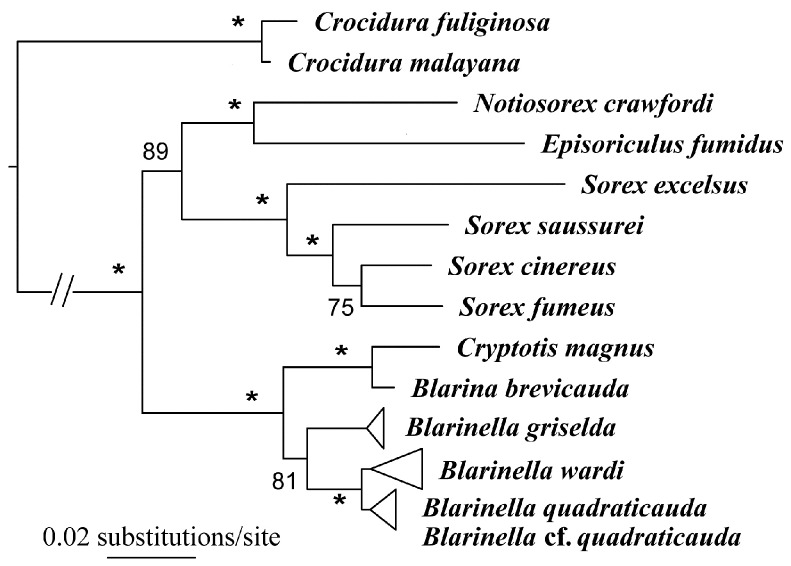
*ApoB* gene tree

In our *ApoB* nuclear gene tree, the same interspecific relationships were recovered in Blarinini-Blarinellini (Figure 3). A sister relationship between Blarinini and Blarinellini was strongly supported (BS=100), and two deeply diverged clades were recovered within *Blarinella* (BS=81). One comprised *B. griselda* from Shaanxi and Gansu. In the other clade, *B. wardi* again occupied a position (BS=97) sister to the remaining samples (i.e., *B. quadraticauda*).

Branch lengths represent substitutions per site. Numbers above branches are bootstrap values supporting the relationship. Asterisks indicate bootstrap value is higher than 90. We collapsed each species branch because of low intra-specific variation.

**Figure 4:**
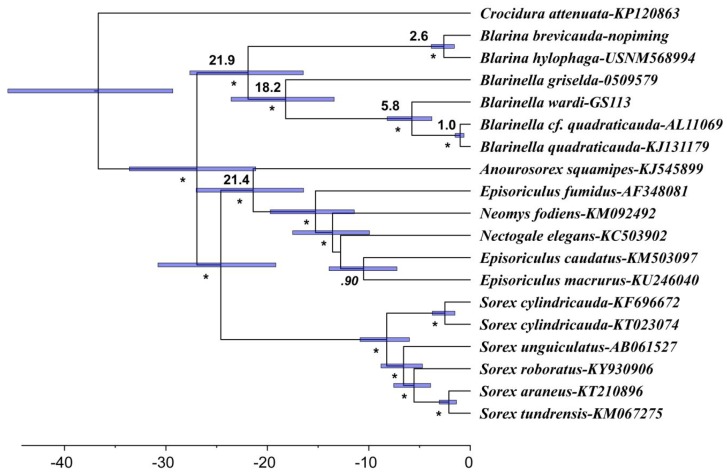
Divergence times estimated using BEAST based on mitogenome data

Our BEAST analyses recovered the same topology (Figure 4) as our mitochondrial and nuclear gene trees. All relationships were strongly supported with posterior probabilities ≥0.98, except for the sister-relationship between *Episoriculus caudatus* and *E. macrurus*, which was 0.90. The divergence time between Blarinini and Blarinellini was estimated to be 21.9 Ma (95% CI=16.5–27.6 Ma), close to that estimated for Anourosoricini and Nectogalini (21.4 Ma, 95% CI=19.2–30.8 Ma). The estimated divergence time between *B. griselda* and other *Blarinella* species was 18.2 Ma (95% CI=13.4–23.6 Ma). The most recent common ancestor of *B. quadraticauda* and *B. wardi* was estimated at 5.8 Ma (95% CI=3.8–8.2 Ma).

Branch lengths represent time. Number below each node denotes posterior probabilities (PP). Asterisks indicate PP is higher than 0.98. Numbers above branches refer to divergence time in millions of years.

### Morphometric comparison

**Figure 5:**
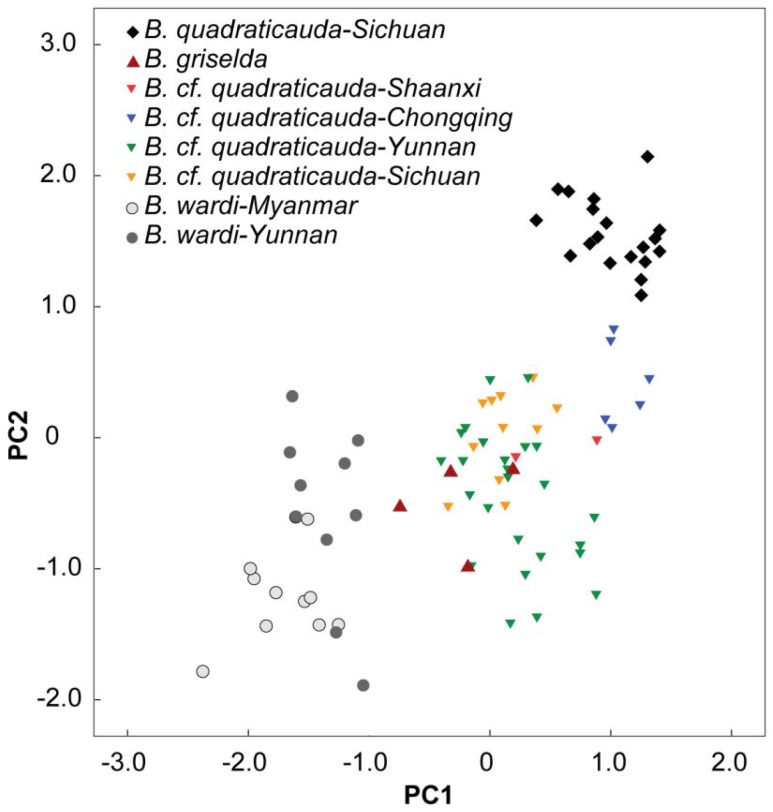
Plot of PC1 and PC2 scores from principal component analyses (PCA) of 19 log_10_-transformed cranial measurements from 87 *Blarinella* specimens

External morphology and skull measurements are given in Table 1. Based on the PCA using craniomandibular measurements of intact skulls, the 1^st^ principal component (PC1) accounted for 72.2% of the variation (eigenvalue=13.7), being positively correlated with all variables (loading >0.69), thus reflecting a size effect. The 2^nd^ principal component (PC2) accounted for 7.3% of the variation (eigenvalue=1.4) and was positively correlated with condyle-glenoid length (CGT), postpalatal length (PPL), and postrostral length (PRL), and negatively correlated with interorbital breadth (IOB), thus indicating strong correlation with the shape of the posterior cranium. On the PC1 and PC2 plot (Figure 5), *B. griselda* overlaps with *B*. cf. *quadraticauda* specimens (i.e. those previously identified as *B. griselda*), suggesting they do not differ from each other by size or overall shape of the skull. When looking at the remaining specimens, *B*. *quadraticauda* occurs along the positive regions of PC1 and PC2, whereas *B*. *wardi* plots on the negative regions of both PC1 and PC2. Furthermore, *B*. cf. *quadraticauda* exhibits geographic variations. For example, the specimens from Chongqing are closest to *B*. cf. *quadraticauda* on the plot, being distinguishable from the specimens from Yunnan, Sichuan, and Shaanxi. In addition, *B. wardi* from Yunnan and northern Myanmar also diverge, although they still overlap on the plot.

**Table 1:** External and craniomandibular measurements, including mean values, standard deviations (top line), together with range and sample sizes (bottom line) of species included in the current study

Variable	*B. griselda*	B. *quadraticauda*	B. cf. *quadraticauda*	B. *wardi*
HB	66.33±0.47	72.21±4.01	65.19±4.32	64.04±2.77
	66–67; 3	65–81; 19	52–73; 47	60–69; 23
TL	36.67±1.7	45.63±4.13	36.37±2.38	37.57±3.14
	35–39; 3	40–60; 19	31–39; 46	32–43; 21
HF	11±0	14.53±0.99	11.04±1.53	11.83±0.52
	11–11; 3	13–16; 19	8.5–13; 46	10.5–13; 23
CIL	19.52±0.34	21.32±0.18	19.97±0.42	19.15±0.38
	19.14–19.96; 4	21–21.69; 19	19.13–20.93; 47	18.54–19.91; 23
IOB	4.33±0.13	4.45±0.15	4.39±0.2	4.04±0.09
	4.2–4.52; 4	4.13–4.66; 19	4.06–4.83; 47	3.89–4.33; 23
CB	9.08±0.26	9.5±0.1	9.12±0.22	8.33±0.22
	8.85–9.48; 4	9.34–9.7; 19	8.57–9.63; 46	7.84–8.72; 22
CH	5.52±0.52	5.38±0.15	4.98±0.22	4.58±0.2
	4.62–5.89; 4	5.11–5.82; 19	4.43–5.15; 46	4.12–4.93; 23
RL	6.51±0.15	7.68±0.25	7.25±0.35	6.64±0.2
	6.32–6.73; 4	7.19–8.05; 19	6.79–7.96; 47	6.25–7.11; 23
PRL	11.49±0.28	12.59±0.2	11.81±0.31	11.58±0.29
	11.2–11.96; 4	12.28–12.94; 19	11.26–12.51; 46	11.06–12.11; 23
MB	5.78±0.18	6.14±0.12	5.91±0.15	5.39±0.15
	5.57–6.05; 4	5.91–6.36; 19	5.62–6.26; 46	5.04–5.77; 23
PIL	8.62±0.19	9.49±0.09	8.93±0.24	8.42±0.22
	8.42–8.9; 4	9.33–9.66; 19	8.26–9.45; 47	7.93–8.78; 23
PPL	9.15±0.29	9.95±0.17	9.28±0.23	9.15±0.25
	8.76–9.5; 4	9.54–10.25; 19	8.7–9.76; 46	8.67–9.63; 23
UTR	8.34±0.16	8.74±0.11	8.34±0.24	7.63±0.24
	8.16–8.59; 4	8.37–8.88; 19	7.79–8.64; 47	6.98–7.97; 23
M^2^-M^2^	5.25±0.11	5.51±0.13	5.29±0.12	4.81±0.11
	5.15–5.44; 4	5.34–5.77; 19	5.08–5.58; 47	4.56–5.03; 23
ML	10.27±0.21	10.7±0.15	10.06±0.32	9.52±0.29
	10.04–10.61; 4	10.37–11; 19	9.39–10.86; 46	8.91–9.95; 22
LTR	7.83±0.16	8.6±0.1	8.1±0.2	7.5±0.16
	7.66–8.07; 4	8.41–8.77; 19	7.55–8.47; 46	7.08–7.74; 22
LLI	4.49±0.07	5.21±0.14	4.73±0.2	4.39±0.18
	4.4–4.6; 4	4.84–5.49; 19	4.2–5.04; 46	4.13–4.79; 22
CGT	8.69±0.17	9.27±0.14	8.74±0.18	8.61±0.2
	8.54–8.96; 4	9.04–9.55; 19	8.36–9.14; 46	8.13–8.96; 23
BCP	1.19±0.01	1.34±0.05	1.2±0.09	1.11±0.09
	1.18–1.2; 4	1.24–1.45; 19	1.03–1.4; 46	0.98–1.34; 22
MTL	4.73±0.15	5.23±0.08	5.03±0.11	4.68±0.11
	4.58–4.92; 4	5.12–5.41; 19	4.8–5.29; 47	4.37–4.87; 23
PPL	3.32±0.08	3.64±0.08	3.47±0.1	3.21±0.08
	3.23–3.44; 4	3.48–3.78; 19	3.29–3.65; 47	3.07–3.34; 23
ZP	2.03±0.09	2.28±0.09	2.14±0.1	1.97±0.11
	1.91–2.14; 4	2.16–2.44; 19	1.86–2.38; 47	1.7–2.2; 23

*B*. cf. *quadraticauda* represents specimens originally identified as *B. griselda*. Abbreviations of variable names are explained in the Materials and Methods. All measurements are in mm.

Most specimens were previously examined by Jiang et al. (2003). Specimens identified as *B*. cf. *quadraticauda* were originally identified as *B. griselda* in Jiang et al. (2003).

### Morphological diagnosis

**Figure 6:**
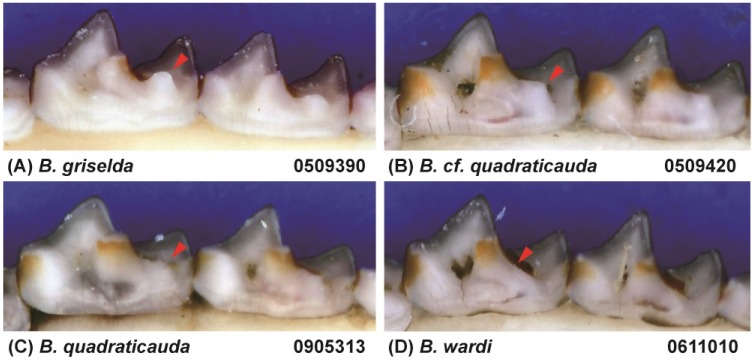
Buccal view of lower M_1_ and M_2_ of four Blarinellini specimens

**Figure 7:**
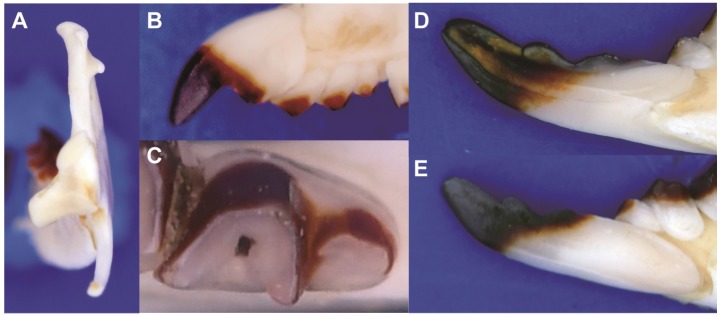
Craniodental characters of *B. griselda*

Because of the deep genetic divergence between *B. griselda* and the other *Blarinella* taxa, it is necessary to describe the morphology at higher taxonomic levels. *Blarinella griselda* exhibits entoconid crests on the lower M_1_ and M_2_ (Figure 6A), which are missing in Blarinini; its mandibular condyle also has a broad interarticular area (Figure 7A), which differs from Anourosoricini and Nectogalini, both of which have a narrow interarticular area; *B. griselda* has large but not fissident upper incisors (Figure 7B), which differs from Beremendiini with strongly fissident upper incisors. Finally, the M_3_ of *B. griselda* has a highly reduced talonid (Figure 7C), which differs from Soricini and Notiosoricini.

Interspecific variations among entoconids and entoconid crests. The arrows point at the entoconids of M_1_. In *B. wardi*, entoconid does not present.

Articular facets of mandibular condyle (A), first upper incisor (B), lower M_3_ (C), buccal view (D) and lingual view (E) of lower incisor.

The craniodental morphology of *B. griselda* closely matches descriptions given by Reumer (1998) for the tribe Blarinellini (see Systematic Biology section) with a single exception regarding the position of the entoconid and shape of the entoconid crest on the lower M_1_ and M_2_, which are variable in *Blarinella* (Figure 6), as discussed in a previous study (Storch, 1995).

**Figure 8:**
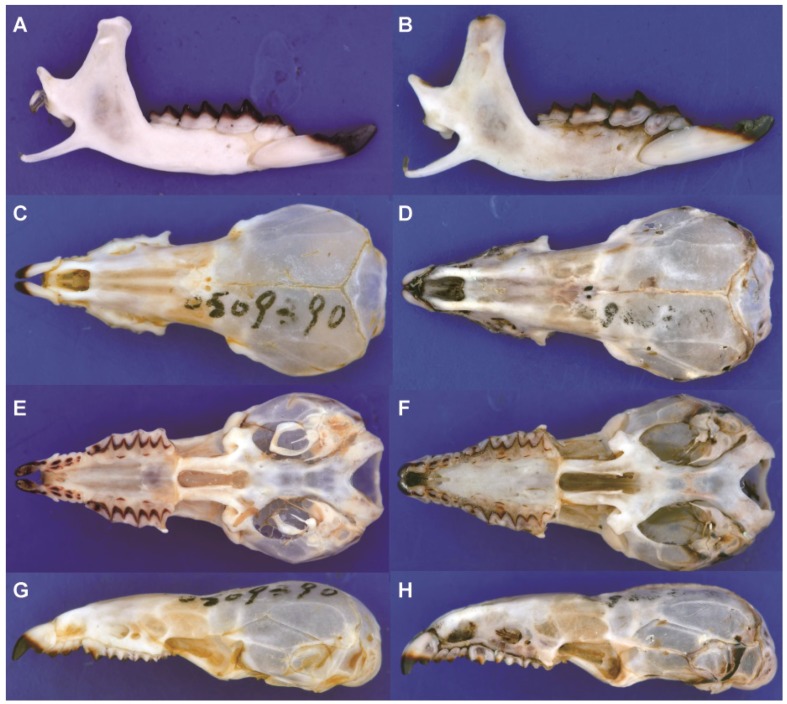
Mandibles and skulls of *B. griselda* (KIZ0509390; A,C,E,G) and *B. quadraticauda* (KIZ0905313; B,D,F,H)

**Figure 9:**
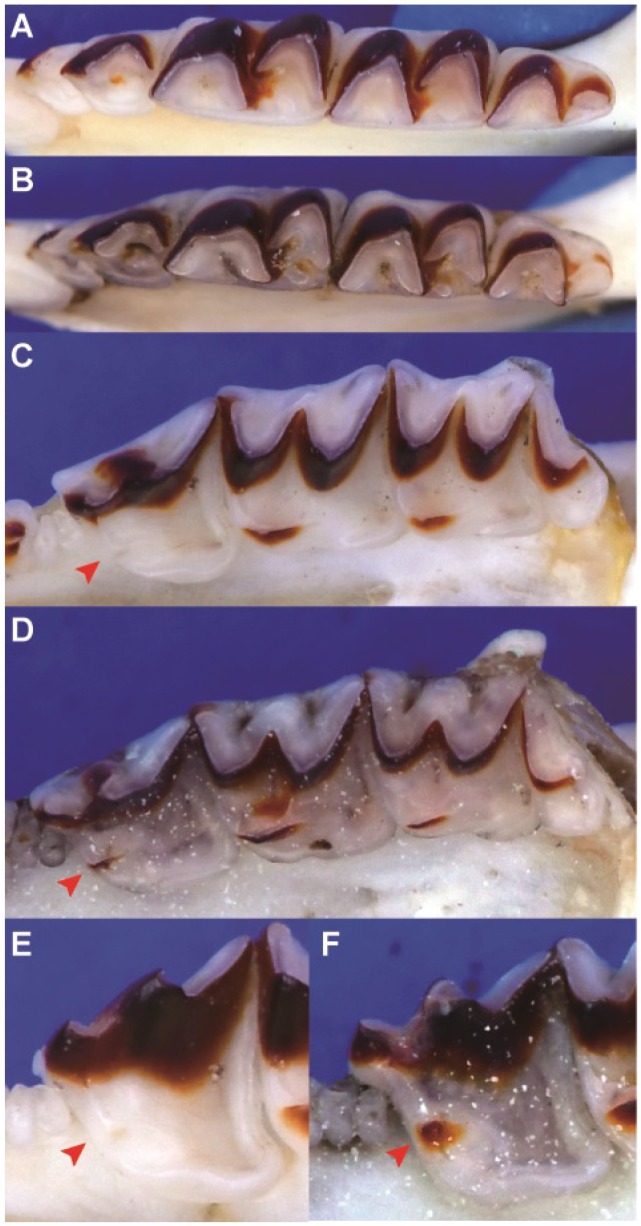
Occlusal views of lower teeth and upper teeth, and occlusal view of upper P^4^ of *B. griselda* (A,C,E) and *B. quadraticauda* (B,D,F). Arrows point at the protocone of upper P^4^

Though *B. griselda* does not differ from *B.* cf. *quadraticauda* morphometrically (see Figure 5), there are several characters of the skull and teeth that differentiate *B. griselda* from *B*. *quadraticauda*, *B*. cf. *quadraticauda*, and *B.* *wardi*. For example, the tip of the coronoid of *B. griselda* is bent anteriorly relative to its position in the other species (Figure 8). The lambdoid crest (dorsal view) of *B. griselda* is triangle-shaped, whereas in the other species the crest is arcuate, extending more anteriorly (Figure 8). The lower P_4_ of *B. griselda* is small, whereas P_4_ extends anteriorly in other species (Figure 9A). On the lower molars of the other species, the paraconids extend anteriorly and the protoconids extend buccally, so that the trigonid basin area is large. Conversely, the paraconids and protoconids of *B. griselda* are not extended, and thus the trigonid basin is smaller (Figure 9A). In *B. griselda*, the entoconid and metaconid on M_1_ and M_2_ are close to each other but well separated, while the entoconid crests are present but distinctly lower than these two cusps (Figure 6A). In the other species, the entoconid and metaconid are either close to one another, connected by a high crest (*B. quadraticauda*; Figure 6B, C), or the entoconid is inconspicuous (*B. wardi*; Figure 6D), although there are some exceptions in *B. quadraticauda*. On unworn upper incisors, the apex of *B. griselda* extends more anteriorly, whereas the apex extends downward in the other species (Figure 8). The upper P^4^ of *B. griselda* has a triangular occlusal outline (Figure 9C), whereas P^4^ in the other species has a quadrangular occlusal outline (Figure 9D). These nuanced but stable characteristics distinguish *B. griselda* from *B. quadraticauda*, *B.* cf. *quadraticauda,* and *B. wardi*. In consideration of the deep genetic divergence and morphological distinctions, we elevate *B. griselda* to a new genus, as described below.

### Systematic biology

Family Soricidae G. Fischer, 1814

Subfamily Soricinae G. Fischer, 1814

Tribe Blarinellini Reumer, 1998

The tribe was defined by Reumer (1998) basically based on dental morphology: “*horizontal ramus of mandible short and high, making the lower dentition compressed anteroposteriorly, and giving the lophs and lophids a compressed W-shaped appearance, mandibular condyle large, with a broad interarticular area; coronoid spicule of mandible well developed; teeth heavily pigmented; upper incisor protruding but not fissident; upper molariform teeth with a reduced posterior emargination, showing a tendency to develop a continuous endoloph; occlusal surface of M_1_ nearly square; M_3_ with a reduced talonid*”. The “lower molars with the entoconid close to the metaconid so that the entoconid crest is short and high” are variable in *Blarinella* (Figure 6), as observed in a previous study (Storch, 1995).

### *Pantherina* He Kai, gen. nov.

**Type species**: *Pantherina griselda* (Thomas, 1912)

**Etymology**: The genus name derives from the feminine Latin noun *Panthera* (“panther”) + the diminutive suffix *-ina*, hence, “little panther”. The name indicates animals in this genus are small but as black and aggressive as a panther. Griselda is a feminine given name.

Suggested common name: Panther shrew; 豹鼩属.

Included species: Type species only.

**Diagnosis**: External morphology and overall shape of skull very similar to *Blarinella*. Tip of coronoid process slightly bends anteriorly, differs from *Blarinella* (continuing vertically). Lambdoid crest angular in dorsal view (more rounded in *Blarinella*). Five upper unicuspids (four upper unicuspids in *Petenyia*). I_1_ bicuspulate, with minute third posterior cuspule present (tricuspulate in *Alloblarinella* and *Zelceina*). Lower P_4_ small, not extending anteriorly (larger and extending anteriorly in *Blarinella*). Trigonid occlusal outline of lower molars trapezoidal-shaped (V-shaped or U-shaped in *Blarinella*). Apex of upper incisors extends anteriorly (directly downward in *Blarinella*). Upper P^4^ with triangular occlusal outline; protocone low, forming antero-lingual corner on ventral outline (trapezoidal-shaped in *Blarinella*). 

**Description**: Skull and mandible: Skull stout and robust. Rostrum short. Braincase dome-shaped but low. Occipital bone small, lambdoid crest triangle-shaped from dorsal view. Entoglenoid processes well developed. Horizontal ramus high. Coronoid process broad and spatulate-shaped, tip of coronoid bends anteriorly, anterior edge of coronoid concave. Coronoid spicule high and strongly pronounced, external temporal fossa shallow. Articular facets of condyle close to each other, with angle of intersection approximately 45º, upper facet narrow and cylinder-shaped, interarticular area broad.

Lower teeth: Lower incisor (I_1_) obviously bicuspulate, with minute third posterior cuspule present. On buccal side, posterior end reaches level of anterior part of M_1_. On lingual side, slender groove obviously presents along I_1_ toward tip. First lower unicuspid very small, larger part of tooth squeezed between I_1_ and P_4_. Occlusal outline of P_4_ bulbous-shaped. Trigonids of M_1_ and M_2_ trapezoidal-shaped. On M_1_, M_2_, entoconids high and close to metaconid, with two cusps connected by low entoconid crest. M_3_ with trigonid unreduced in size but with talonid reduced to single, low, nearly conical, slightly blade-like hypoconid, with no trace of entoconid crest.

Upper teeth: Upper I^1^ noticeably extends anteriorly. Apex of upper I^1^ moderately long and broad, spatulate, not fissident; talon lower than first upper unicuspid. Five upper unicuspids (U) present. From occlusal view, U^1^–U^5^ decreases in size of total area in order. U^1^ twice as large as U^2^, and U^4^ and U^5^ extremely small. U^1^–U^4^ visible in lateral view of skull. P^4^ with no posterior emargination; protocone small, forming antero-lingual corner in outline of tooth; hypocone absent, hypoconal flange extends to approximately level of metacone; occlusal outline of P^4^ triangular in shape. M^1^ and M^2^ similar in shape: protocone low, forming antero-lingual corner in ventral outline, hypocone absent, hypoconal flange extends posteriorly; occlusal outline square-shaped. M^3^ small, with well-developed paracone, lingual part consists of basin surrounded by U-shaped ridge.

**Remarks:** Some characters observed in *P. griselda* are more similar to fossil genera related to extant *Blarinella* species. For example, the anterior projection of the tip of the coronoid process in *P. griselda* has also been observed in *Petenyia hungarica* and *Zelceina kormosi* (Reumer, 1984; Storch, 1995). The entoconid and entoconid crest on the lower M_1_ and M_2_ of *P. griselda* have been observed in *Petenyia hungarica* (Reumer, 1984). Of note, this is a variable character in *Blarinella*, with some *B. quadraticauda* also having a similar structure. The triangular-shaped P^4^ of *P. griselda* is somewhat similar to that of *Alloblarinella sinica* and *Petenyia hungarica* and quite similar to that of *Petenyia bubia* (Reumer, 1984).

Pantherina griselda

*Blarinella griselda* (Thomas, 1912)

*Blarinella quadraticauda griselda* (Allen, 1938)

**Type locality**: 68 km SE Taochou (=Lingtan), 3 048 m a.s.l., Gansu, NW China.

**Holotype**: Natural History Museum (British) No. 12.8.5.23, collected by J.A.C. Smith

Suggested common name: Gray panther shrew; 淡灰豹鼩

**Measurements**: Table 1

Figure 6A, Figure 7, Figure 8 left, Figure 9A, C, E

**Distribution**: Currently known from two localities in southern Gansu and southern Shaanxi (Figure 1).

**Description**: As for the genus.

**Comparison**: Specimens of *Pantherina griselda* from Shaanxi are superficially similar to *Blarinella* in gross morphology; however, a number of characters distinguish the two taxa. In *P. griselda*, the lambdoid crest is angular in dorsal view, whereas in species of *Blarinella*, the occipital bone extends anteriorly, resulting in a more rounded lambdoid crest (Figure 8B). The tip of the coronoid process projects anteriorly rather than continuing vertically, as in *Blarinella*. The groove on the buccal side of I_1_ is narrow, rather than broad in *Blarinella*. In the labial view, P_4_ of *P. griselda* does not extend anteriorly, so the tooth and trigonid basin are smaller than that of *Blarinella*, in which the P_4_ extends anteriorly. In *P. griselda*, the paraconids of M_1_ and M_2_ do not extend anteriorly, the trigonid occlusal outline is trapezoidal, and the trigonid basin area is small, whereas in *Blarinella*, the paraconids of M_1_ and M_2_ extend anteriorly, the metaconid extends posteriorly, the trigonid has a V-shaped or U-shaped occlusal outline, and the trigonid basin area is large. In *P. griselda*, the entoconid and metaconid on M_1_ and M_2_ are high and connected by distinctly lower entoconid crest. *Blarinella wardi* has no entoconid or entoconid crest on M_1_ or M_2_, but the posterior faces of its metaconids extend posteriorly forming a posterior ridge, which is not present in *Pantherina*. In *B. quadraticauda* (including *B.* cf. *quadraticauda*), the entoconid crest is variable, depending on the locality. For example, some specimens from the type locality (i.e., northwestern Sichuan) have a low, moderately-developed entoconid located close to the metaconid, resulting in a short and moderately-high entoconid crest; in a few populations from northeastern Yunnan (*B.* cf. *quadraticauda*), the entoconid and entoconid crest are more similar to those of *P. griselda,* although the height of the entoconid is lower; in other samples, the entoconid is not obviously present and is more similar to *B. wardi*. The upper I^1^ of *P. griselda* extend anteriorly, with a long apex, whereas, in *Blarinella*, the apex is shorter and typically directed ventrally. The occlusal outline of P^4^ in *P. griselda* is triangular and the protocone is small, forming an antero-lingual corner in the outline of the tooth, whereas in *Blarinella*, the occlusal outline of P^4^ is mostly trapezoidal, and the protocone is more robust, not forming an antero-lingual corner in the outline. On the M^1^ and M^2^ of *P. griselda,* the hypoconal flange extends posteriorly, whereas in *Blarinella*, the hypoconal flange extends anteriorly.

## DISCUSSION

### Taxonomic implications

Our results strongly support the conclusion that specimens ascribed to *Blarinella griselda* are paraphyletic; one is a close relative to *B. quadraticauda*, whereas the other presents an anciently diverged lineage of Blarinellini. The divergence time estimation (18.2 Ma) suggests a divergence more ancient than the ancestor of Nectogalini, and close to that between Anourosoricini and Nectogalini. Morphometric analyses could not differentiate the two forms, indicating that phenotypic evolution was not strongly associated with quantitative characters. However, several nuanced but stable diagnostic characters on the skull and dentition distinguish *B. griselda* from other species of *Blarinella*. Some of these craniodental differences were observed for several fossil genera of the tribe. In other words, the difference between *B. griselda* and members of the genus *Blarinella* is at the same level as that between currently recognized genera. Because the two extant lineages showed marked genetic divergence from one another (Figure 2, Figure 3), and because of the observation of inter-generic level morphological variation (Figure 6, Figure 7, Figure 8 and Figure 9), we recognize *B. griselda* as a distinct genus, namely *Pantherina*.

It should be noted that *Pantherina* shares several characters with fossil taxa, such as a triangle-shaped P^4^ on occlusal view, indicating that the genus represents a more primitive form of the tribe and may have closer affinities with fossil taxa within Blarinellini. We have not attempted to fully revise the systematic paleontology in the current study, but taxonomic revision that includes fossils is warranted. We do not currently recognize *Pantherina* as one of the fossil genera based on the obvious differences in the skull and dentation. For example, it should not be assigned to *Alloblarinella* or *Zelceina* whose lower incisor is tricuspulate (bicuspulate in *Pantherina*). It should not be assigned to *Petenyia* which has only four unicuspids (*Pantherina* has five). Its small M_3_ also differs from that of *Alloblarinella* which has a large M_3_ with complete trigonid and talonid.

The new monotypic genus *Pantherina* is known currently only from southern Gansu (Lintan) and southern Shaanxi (Ningshaan). Despite extensive recent surveys (Chen et al., 2012; K.H., unpublished data), members of this genus have not been found in southern areas of China such as Chongqing, Hubei, or northwestern Sichuan—areas that are, however, occupied by *B. quadraticauda*. *Pantherina* is unlikely to be distributed in more northern areas due to absence of suitable habitat, and thus presumably has a restricted distribution in southern Shaanxi and southern Gansu. Because *Pantherina* has similar morphology to *B. quadraticauda*, and because they co-occur in Shaanxi, they might compete for the same habitat.

We tentatively assign the remaining specimens previously identified as *B. griselda* from China and Vietnam to *B.* cf. *quadraticauda* because they clustered together with *B. quadraticauda* in a well-supported clade in both the mitochondrial (Figure 2) and nuclear gene trees (Figure 3), with the latter forming a lineage well embedded within this clade (Figure 2). This placement does not mean that a two-species scenario (*B. quadraticauda* and *B. wardi*) is sufficient to cover the species diversity of this genus. Instead, we believe that species diversity is still underestimated, as implied in our morphometric and phylogenetic analyses. For example, on the PCA plot, the animals from Chongqing are clearly distinguished from other *B. quadraticauda* (Figure 5). The mitogenomic gene tree supports multiple fine-supported subclades within *B. quadraticauda* (i.e., BS≥70), which also show a strong geographic pattern. The animals from Shaanxi and northeastern-most Sichuan cluster together, forming a basal divergence within *B. quadraticauda* (BS=73). The specimens from Vietnam are also of two different originations (BS=100). Whether these distinguishable clades or morphometric groups represent undescribed species/subspecies or geographic populations remain open questions.

### Implications for systematics and macroevolution

Recognition of a new genus of Blarinellini is especially interesting, not only because it is the second extant genus of the tribe, but because it could provide clues to fill the evolutionary relationship gaps among living and fossil taxa of this genera-rich tribe. Reumer (1998) assigned *Blarinella* together with eight fossil genera into Blarinellini, and *Tregosorex*, previously assigned to Blarinini, has also been assigned recently based on diagnosis of newly discovered fossil material (Doby, 2015). With the addition of *Pantherina*, 11 genera can be recognized, which is the largest number for the Soricinae. The genus boundaries of Blarinellini shrews were established exclusively based on morphological diagnosis and comparison, and some species have been moved from one genus to another. The current classification and systematic hypotheses for the tribe Blarinellini are difficult to test using molecular-based approaches because there are no available resources. Although it is possible to use a morphological matrix, the results may be affected by homoplasy, especially when the number of characters is not large and molecular data are not integrated. Indeed, phylogenic analysis was carried out for Eurasian soricine shrews to include six genera of Blarinellini but revealed five polyphyletic lineages (Rofes & Cuenca-Bescos, 2009). Because all characters were equally weighted (which is usually not a good assumption for morphological characters), the topology was not constrained, and molecular data were not incorporated, high-level relationships among extant taxa were very different from the well-known molecular phylogenies: *Blarinella* was grouped with Anourosoricini (which is closely related with Nectogalini; see Figure 2), and *Neomys* (a nectogaline genus) was not grouped with the other Nectogalini shrews (see Figure 2). As acknowledged by the authors, the homoplasy index was high, so it may suffer convergence in morphology (Springer et al., 2013), which is very common in semi-fossorial small mammals (He et al., 2015). These previous studies suggest that a systematic relationship exclusively based on morphological data may be biased. The time-calibrated tree accomplished by a morphological matrix could provide a scaffold for analyzing craniodental evolution in the tribe, thereby assisting in examining hypotheses of systematic relationships. The latter could be especially true because *Pantherina* seems to represent a more primitive lineage than *Blarinella*, as some characteristics of *Pantherina* are also observed in fossil taxa. These include a more triangle-shaped P^4^, which is similar to that of *Petenyia bubia* from the Miocene. The presence of entoconids and entoconid crests on the lower M_1_ and M_2_ also seem to be pleiomorphic, which has been observed in many primitive fossil shrews, including Heterosoricinae from the Eocene (Repenning, 1967). Among the fossil genera of Blarinellini, four were exclusively distributed in Eurasia (*Alloblarinella*, *Cokia*, *Hemisorex*, and *Paenepetenyia*) and four were exclusively from North America (*Alluvisorex*, *Anchiblarinella*, *Parydrosorex*, and *Tregosorex*). In addition, *Petenyia* was distributed throughout both North America and Eurasia. Finely-resolved phylogenetic and systematic relationships could help to illustrate the pattern of transcontinental migrations through time (Dubey et al., 2007).

We are grateful to Esther Langan and Darrin Lunde, National Museum of Natural History, Smithsonian Institution, and Robert Voss and Guy Musser, American Museum of Natural History, for allowing us to access the specimens under their care. We are also grateful to Esther Langan and Darrin Lunde for providing the tissue samples of *Blarina hylophaga*. We are grateful to Margaret Docker for allowing us to use ION Torrent PGM in her lab. We thank the anonymous reviewers for positive comments and suggestions and thank Neal Woodman for his suggestions and extensive editing for improvement. We also thank Qiang Li and Yi-Kun Li from the Institute of Vertebrate Paleontology and Paleoanthropology, Chinese Academy of Sciences for sharing literature.

## Appendix I

Specimens examined.

Abbreviations: KIZ: Kunming Institute of Zoology, Chinese Academy of Sciences, AMNH: American Museum of Natural History, USNM: National Museum of Natural History, Smithsonian Institution.

*B. quadraticauda* (*n*=19)

Baoxin, Sichuan (KIZ820495, KIZ820588, KIZ820625, KIZ820626),

Qionglai, Sichuan (AMNH111121, AMNH111125, AMNH111126, AMNH111127, AMNH111128, AMNH111130, AMNH111136)

Wenchuan, Sichuan (AMNH111112, AMNH111114, AMNH111119, AMNH111137, AMNH111138, AMNH111140, AMNH111142, AMNH111145)

*B. cf. quadraticauda* (identified as *B. griselda* in Jiang et al., 2003; *n*=45)

Jingdong, Yunnan (KIZ640068, KIZ640105, KIZ640128, KIZ640129, KIZ640133, KIZ640134, KIZ640152, KIZ640191, KIZ640199, KIZ640200, KIZ640418, KIZ640429, KIZ640430, KIZ640431, KIZ640432, KIZ96180, KIZ98069, KIZ98107, KIZ98114, KIZ98151, KIZ98152, KIZ98313, KIZ98335, KIZ98378, KIZ98553, KIZ72053), Luchun, Yunnan (KIZ72053), Mt. Qinling, Shaanxi (KIZ0509420, KIZ0509423), Nanchuan, Sichuan (KIZ88126, KIZ88127, KIZ88252), Shimian, Sichuan (KIZ820742, KIZ820743, KIZ820744, KIZ820752, KIZ820753, KIZ820754, USNM574289, USNM574290, USNM574291, USNM574292, USNM574293), Wulong, Sichuan (KIZ88320, KIZ88321, KIZ88395)

*B. griselda* (*n*=4)

Gansu (AMNH60449), Mt. Qinling, Shaanxi (KIZ0509390, KIZ0509578, KIZ0509579)

*B. wardi* (*n*=25)

Bijiang, Yunnan (KIZ820280), Deqin, Yunnan (KIZ820280), Gongshan, Yunnan (KIZ73863, KIZ73889, KIZ73910, KIZ73920, KIZ73927, KIZ73928, KIZ73929), Lijiang, Yunnan (USNM241403), Myanmar (AMNH114720, AMNH114721, AMNH114722, AMNH114723, AMNH114739, AMNH114740, AMNH114741, AMNH114748, AMNH114757, AMNH114758, AMNH114760), Pianma, Yunnan (KIZ74176), Weixi, Yunnan (KIZ810669), Yinjiang, Yunnan (KIZ76286), Zhongdian, Yunnan (KIZ810147)
